# Including migrant populations in health impact assessments

**DOI:** 10.2471/BLT.14.142315

**Published:** 2015-11-02

**Authors:** Lara Miramontes, Kevin Pottie, Maria Benkhalti Jandu, Vivian Welch, Keith Miller, Megan James, Janet Hatcher Roberts

**Affiliations:** a Simon Fraser University Faculty of Health Sciences, Burnaby, Canada.; b WHO Collaborating Center for Knowledge Translation and Health Technology Assessment in Health Equity, Centre for Global Health, University of Ottawa, Bruyère Research Institute, 43 Bruyère Street, Ottawa, K1N 5C8 Canada.; c Institute of Population Health, University of Ottawa, Ottawa, Canada.; d Bruyère Research Institute, University of Ottawa, Ottawa, Canada.

In 2010, there were 214 million international migrants worldwide, a number that is projected to double by 2050.^1^ Migrants’ motives for leaving their countries of origin include employment and education opportunities, escape from conflict and discrimination and the desire to raise families in economically and politically stable environments.

New migrants are often healthier than the general population on arrival, but their health may deteriorate after settlement,^2^ due to unfamiliar social conditions, infectious diseases, or restricted access to health services. Cultural and linguistic barriers may contribute to poor delivery of health services. The 61st World Health Assembly called on all Member States to “promote migrant-sensitive health policies”.^3^ Some subgroups – especially refugees – have a greater burden of infectious diseases and mental disorders than the indigenous population.^4^ Guidelines have been developed to assist health workers in the clinical management of migrating populations.^4^ However, there are no explicit decision-support tools for policy-makers to ensure health equity for migrants. Here we discuss how health impact assessment can account for the needs of migrant populations.

## Health impact assessment

Health impact assessment can improve health equity by mitigating unintended harms and maximizing the benefits of programmes or policies. This approach supports decision-makers by suggesting actionable recommendations for emerging policies and programmes.^5^ A number of health impact assessment tools have been developed,^6^ several of which have emphasized the need to consider disadvantaged population groups. However, a recent review found that only 14% of health impact assessments mentioned migrants in the evaluation and only 2% included them in their recommendations.^7^ A recent consultation held at the WHO Collaborating Centre for Knowledge Translation and Health Technology Assessment in Health Equity identified four challenges to the inclusion of migrants in health impact assessment: (i) including migrants in the scope of the assessment; (ii) obtaining data on migrants; (iii) engaging migrant communities; and (iv) successfully appealing to decision-makers.

## Addressing the challenges

Based on our own experience from developing migrant-focused guidelines in Canada, we discuss how these four challenges can be addressed.

The first challenge is to include migrants in the scope of the assessment. Ideally, scoping should be deliberate and involve health researchers and community experts. In practice, however, the scope of health impact assessment is limited by timelines, political agendas and resources. Those who perform health impact assessments may only have the capacity to consider accessible populations and this may leave out migrants and other vulnerable populations. When appropriate, irregular migrants and/or refugee populations should be included in health impact assessments to maximize the value of the assessment and its recommendations. Appropriate scenarios for inclusion would include existing or future risk for inequities in migrant populations.

Equity considerations should be consistent and well-defined. For example, some studies may erroneously consider ethnicity and migrant status as interchangeable. Migration, in itself, may be a unique social determinant of health distinct from ethnicity, language, culture or religion. Vulnerable subgroups of the population should be integrated within health system policies and programmes, with consideration given to historical contexts and local capacity, such as universal access to basic health care. Without explicit mention of migrants during the scoping stage, migrant populations may be forgotten in the assessment and recommendations.

The second challenge is to source migrant-specific data. Migrants are heterogeneous, in terms of countries of origin, migration experiences, status, needs and abilities. Some migrant populations may be available or more readily accessible for data collection, while temporary workers, refugees and irregular migrants may be less accessible. Organizations and advocates for migrant communities can provide data on access to services and geographical location of services in relation to migrant populations. For certain groups – such as those with language and literacy limitations – equity extrapolation methods that include adjusting existing estimates using PROGRESS equity factors,^8^ absolute risks and patient values and preferences may be required.^4^ Some migrants – such as those that have entered a country without official migration status – may be apprehensive of authority and data collection procedures or may be afraid to access health care and other services.

Health impact assessment should be evidence-based, but data that are specific to migrants may not be available. To address this gap, assessment practitioners could use international and regional sources of data – such as evidence-based guidelines on immigrants and refugees,^4^ the Migration Integration Policy Index,^9^ statistics from the International Organization for Migration and data from censuses and community organizations.

The third challenge is to engage migrant communities. The diversity of migrant populations presents a challenge to community consultation and engagement. Many health impact assessment frameworks recommend ensuring significant community involvement in the process, but this can be difficult to achieve with migrant populations. Those unfamiliar with working with migrants may find it difficult to attract and retain authentic engagement with these communities. Interpretation, translation and cultural mediation, combined with resources to facilitate participation, such as childcare and transportation, may assist practitioners. Competing interests, ethnic or tribal strife, cultural beliefs such as gender dynamics and loss to follow-up can limit the participation of migrants.

While community engagement is challenging, promising community partnerships are now emerging in many areas – such as Local Immigration Partnerships in many Canadian cities. Such groups can work to identify migrants’ needs and may be a resource for community engagement in health impact assessment. To mitigate language, logistical, cultural and conceptual challenges, those implementing health impact assessments should collaborate with the relevant advocates, agencies and community organizations. These partners may be able to ensure that migrants are considered, engaged and served by the process, as the most valuable stakeholders in their own health and equity outcomes.

The fourth challenge is to make programme and policy recommendations appealing and feasible. Decision-makers may not see the need or political benefit of adapting policies and programmes solely for migrant populations. Assessments that consider all persons affected by migration, as well as migrants and disadvantaged local populations may lead to less polarizing and more sustainable policy recommendations. Exclusively targeting migrants in the assessment may be a politically sensitive choice, especially when disadvantaged migrant groups are seen as a burden for the community. Coordination and collaboration with advocates may be necessary to help decision-makers see the political value of addressing the needs of all people affected by migration. Personal narratives and success stories can be used when adapting programmes and policies to enhance equity. 

In conclusion, several factors contribute to the exclusion of migrant populations from research and decision-making in health impact assessments. We suggest that tools should be developed to assist practitioners to assess subgroups, to engage migrant communities and to appeal to decision-makers. To ensure that programmes and policies are sensitive to the needs of migrants, migrant communities should be included in health impact assessments. The results should be shared with practitioners and communities affected by migration.
